# Efficacy and Safety of Upadacitinib in a Case of Pediatric Acute Severe Ulcerative Colitis and Immune Thrombocytopenia

**DOI:** 10.1093/ibd/izaf139

**Published:** 2025-07-01

**Authors:** Benedetta Bocchini, Massimo Martinelli, Caterina Strisciuglio, Pio Stellato, Maria Teresa Fioretti, Annamaria Staiano, Erasmo Miele

**Affiliations:** Department of Translational Medical Science, Section of Pediatrics, University of Naples “Federico II,” Naples, Italy; Department of Translational Medical Science, Section of Pediatrics, University of Naples “Federico II,” Naples, Italy; Oncohematology Unit, Department of Oncology, Hematology and Cellular Therapies, Santobono-Pausilipon Children Hospital, Naples, Italy; Department of Woman, Child and General and Specialistic Surgery, University of Campania “L. Vanvitelli,” Naples, Italy; Department of Translational Medical Science, Section of Pediatrics, University of Naples “Federico II,” Naples, Italy; Department of Translational Medical Science, Section of Pediatrics, University of Naples “Federico II,” Naples, Italy; Department of Translational Medical Science, Section of Pediatrics, University of Naples “Federico II,” Naples, Italy

**Keywords:** ulcerative colitis, pediatric inflammatory bowel disease, upadacitinib, immune thrombocytopenia

## Case Report

**Figure 1.**
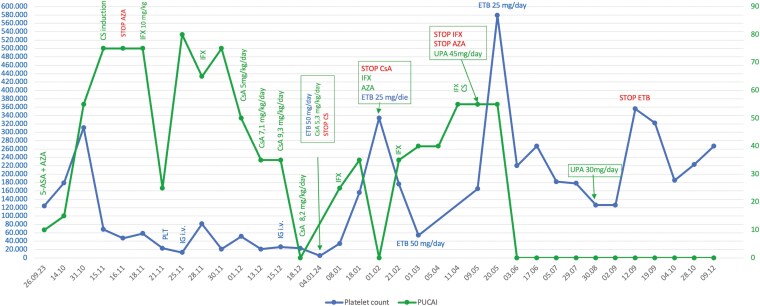
Clinical, laboratory and therapeutical course of a 12 years old girl with an acute severe colitis refractory to conventional therapies. 5-ASA: mesalazine; AZA: Azathioprine; CS: Corticosteroids; CsA: Cyclosporine; ETB: Eltrombopag; IG-iv: Intravenous immunoglobulin; Infliximab: IFX; PLT: platelets; PUCAI: Pediatric Ulcerative Colitis Activity Index; UPA: Upadacitinib.

We report the case of a 12-year-old girl with ulcerative colitis (UC) who had been on treatment with azathioprine (AZA) and 5-ASA for over a year, presenting with a disease flare (PUCAI 55). After an initial improvement following the introduction of antibiotics and methylprednisone, she developed an episode of acute severe colitis (PUCAI 75) associated with thrombocytopenia (68.000 platelets/ul) (Figure 1). Ileocolonoscopy revealed severe pancolitis (E4-S1; UCEIS: 5). Due to steroid failure, second line rescue therapy with infliximab (IFX) at 10 mg/kg was initiated. Thrombocytopenia worsened (23.000/ul), prompting 2 intravenous immunoglobulin (Ig) infusions (0.9 mg/kg) which led to a mild improvement of platelet count (40-50.000/ul). Due to the persistent moderate disease activity, despite the second IFX infusion, and a suspected reduction in IFX efficacy due to the Ig infusions,^1^ oral cyclosporine (CsA) was introduced. Bone marrow evaluation showed a trilinear marrow with well represented erythroid, myeloid and lymphoid lineages along with increased megakaryocyte and features of dysmegakariocytopoiesis, suggestive of immune thrombocytopenia (ITP). Twenty days after second Ig infusion, the platelet count dropped again to 5000/ul. Faced with ITP unresponsive to first line therapies, and in agreement with hematologists off-label therapy with Eltrombopag (50 mg/die) was initiated, yielding an excellent response (334.000/ul). CsA was tapered and fully discontinued after 2 months, when AZA was reintroduced along with IFX infusions every 4 weeks. An attempt to taper Eltrombopag was unsuccessful and due to non-controlled activity (PUCAI 60), despite optimized IFX trough levels, both AZA and IFX were discontinued. Third line therapy with the Jak-1 inhibitor Upadacitinib (UPA), combined with steroids, was initiated. Within 10 days, there was full clinical recovery with normalization of PUCAI, fecal calprotectin and inflammatory markers. Both Eltrombopag and steroids were successfully tapered with UPA maintaining control UC activity and platelet count (Figure 1).

## Discussion

Upadacitinib is a JAK-1 inhibitor approved for treatment of UC in adults. Only a few pediatric case series have been published, demonstrating its efficacy in patients with intractable IBD.^2–6^ Our case supports the potential of UPA as a rescue therapy in children with refractory UC. Additionally, we report ITP as an uncommon extraintestinal manifestation of IBD.^7^ ITP may develop as a result of an immune response against platelet surface peptides that cross-react with bacterial glycoproteins. This immune activation may be triggered by increased mucosal permeability.^8,9^ Indeed, achieving UC remission has been associated with ITP resolution.^10^ Our case further supports this hypothesis, as stabilization of the platelet count occurred only after the resolution of intestinal inflammation following the initiation of UPA.

## Abbreviations

5-ASA: mesalazine
ASC: Acute severe colitis
AZA: Azathioprine
CsA: Cyclosporine
ETB: Eltrombopag
IBD: Inflammatory bowel disease
IFX: Infliximab
PUCAI: Pediatric Ulcerative Colitis Activity Index
UC: Ulcerative colitis
UPA: Upadacitinib
