# Correction to: Efficacy and Safety of Upadacitinib in a Case of Pediatric Acute Severe Ulcerative Colitis and Immune Thrombocytopenia

**DOI:** 10.1093/ibd/izaf245

**Published:** 2025-11-02

This is a correction to: Benedetta Bocchini, Massimo Martinelli, Caterina Strisciuglio, Pio Stellato, Maria Teresa Fioretti, Annamaria Staiano, Erasmo Miele, Efficacy and Safety of Upadacitinib in a Case of Pediatric Acute Severe Ulcerative Colitis and Immune Thrombocytopenia, *Inflammatory Bowel Diseases*, 2025, izaf139, https://doi.org/10.1093/ibd/izaf139

In the originally published version of this manuscript, the affiliations of authors Caterina Strisciuglio and Pio Stellato were inadvertently swapped. This error has been corrected.

